# Epidemiology and diagnostic challenges in neuromyelitis optica spectrum disorder in Taiwan: a hospital-based surveillance accompanied by a nationwide study

**DOI:** 10.1093/braincomms/fcaf293

**Published:** 2025-08-14

**Authors:** Jung Lung Hsu, Jen Jen Su, Mei-Yun Cheng, Ming-Feng Liao, Hung-Chou Kuo, Chun-Che Chu, Chiung-Mei Chen, Kuo-Hsuan Chang, Chun-Wei Chang, Yih-Ru Wu, Chin-Chang Huang, Chyi-Huey Bai, Long-Sun Ro

**Affiliations:** Department of Neurology, New Taipei Municipal TuCheng Hospital (Built and Operated by Chang Gung Medical Foundation), New Taipei City 236017, Taiwan; Department of Neurology, Chang Gung Memorial Hospital Linkou Medical Center and College of Medicine, Chang-Gung University, Linkou, Taoyuan 333423, Taiwan; Taipei Medical University, Graduate Institute of Humanities in Medicine and Research Center for Brain and Consciousness, Shuang Ho Hospital, Taipei 11031, Taiwan; Department of Neurology, National Taiwan University Hospital, Taipei 106319, Taiwan; Department of Neurology, Chang Gung Memorial Hospital Linkou Medical Center and College of Medicine, Chang-Gung University, Linkou, Taoyuan 333423, Taiwan; Institute of Molecular Medicine, National Tsing Hua University, Hsinchu 30013, Taiwan; Department of Neurology, Chang Gung Memorial Hospital Linkou Medical Center and College of Medicine, Chang-Gung University, Linkou, Taoyuan 333423, Taiwan; Department of Neurology, Chang Gung Memorial Hospital Linkou Medical Center and College of Medicine, Chang-Gung University, Linkou, Taoyuan 333423, Taiwan; Department of Neurology, Chang Gung Memorial Hospital Linkou Medical Center and College of Medicine, Chang-Gung University, Linkou, Taoyuan 333423, Taiwan; Department of Neurology, Chang Gung Memorial Hospital Linkou Medical Center and College of Medicine, Chang-Gung University, Linkou, Taoyuan 333423, Taiwan; Department of Neurology, Chang Gung Memorial Hospital Linkou Medical Center and College of Medicine, Chang-Gung University, Linkou, Taoyuan 333423, Taiwan; Department of Neurology, Chang Gung Memorial Hospital Linkou Medical Center and College of Medicine, Chang-Gung University, Linkou, Taoyuan 333423, Taiwan; Department of Neurology, Chang Gung Memorial Hospital Linkou Medical Center and College of Medicine, Chang-Gung University, Linkou, Taoyuan 333423, Taiwan; Department of Neurology, Chang Gung Memorial Hospital Linkou Medical Center and College of Medicine, Chang-Gung University, Linkou, Taoyuan 333423, Taiwan; Department of Public Health and School of Public Health, College of Public Health, Taipei Medical University, Taipei 11031, Taiwan; Department of Neurology, Chang Gung Memorial Hospital Linkou Medical Center and College of Medicine, Chang-Gung University, Linkou, Taoyuan 333423, Taiwan

**Keywords:** neuromyelitis optica, incidence, ARR, comorbidity, misclassification

## Abstract

Neuromyelitis optica spectrum disorder is a rare autoimmune inflammatory demyelinating disease that must be differentiated from multiple sclerosis. The impact of misclassification on these patients in Taiwan remains unclear. We conducted a hospital-based retrospective cohort study of neuromyelitis optica spectrum disorder patients using the Chang Gung Research Database from 2005 to 2021. This cohort included diagnostic serostatus data from anti-Aquaporin 4 antibody testing, providing insights into incidence, demographics, annual relapse rate, and initial misclassification as multiple sclerosis. To extend these findings, we applied the same methodology to a nationwide neuromyelitis optica spectrum disorder cohort (2006–2020) using Taiwan’s National Health Insurance Research Database, which does not include aquaporin 4 serostatus information; thus, the data made it impossible to distinguish seropositive from seronegative nationwide neuromyelitis optica spectrum disorder cases. Misclassification was calculated as the ratio of nationwide neuromyelitis optica spectrum disorder patients initially diagnosed with multiple sclerosis (MS) to the total nationwide neuromyelitis optica spectrum disorder cases in each cohort. In the Chang Gung Research Database cohort, we identified 193 seropositive nationwide neuromyelitis optica spectrum disorder patients, including four (2.1%) paediatric cases. Hospital incidence increased from 0.08 to 1.19 per 100 000 persons (2006–2021). Comorbidities were present in 32% of adults, with Sjögren’s syndrome as the most common (21.2%). Median annualized relapse rate in adults was 0.49 (range, 0.26–0.85) and was higher in the first 3 years post-diagnosis (0.67 versus 0.00; *P* < 0.01). The National Health Insurance Research Database cohort included 1892 neuromyelitis optica spectrum disorder patients, 92 (4.9%) paediatric. Incidence in adults rose from 0.25 to 0.84 per 100 000 persons, with a prevalence of 8.01 per 100 000 in 2020. Median annualized relapse rate was 0.36 (range, 0.07–60.83) for adults and 0.44 (range, 0.07–6.40) for paediatric patients. Misclassification as multiple sclerosis occurred in 55.0% Chang Gung Research Database and 63.5% National Health Insurance Research Database cohort. Misclassified patients were more likely to be younger, female, experience delayed antibody testing, have frequent hospitalizations, and suffer more relapses. Both hospital-based and nationwide cohorts revealed increasing neuromyelitis optica spectrum disorder incidence and prevalence in Taiwan. This early-phase elevation in relapse activity highlights the critical therapeutic window where intervention may most effectively prevent long-term disability. Greater clinical awareness and close follow-up may improve neuromyelitis optica spectrum disorder diagnosis and management.

## Teaser

Hsu *et al*. report the epidemiology and diagnostic challenges of neuromyelitis optica spectrum disorder in Taiwan, highlighting increasing incidence and prevalence, a significant proportion of misclassification, and higher relapse rates within 3 years post-diagnosis. They emphasize the need for early targeted therapies and accurate diagnostics to improve outcomes and reduce disability in neuromyelitis optica spectrum disorder patients.

## Graphical Abstract

**Graphical Abstract:**
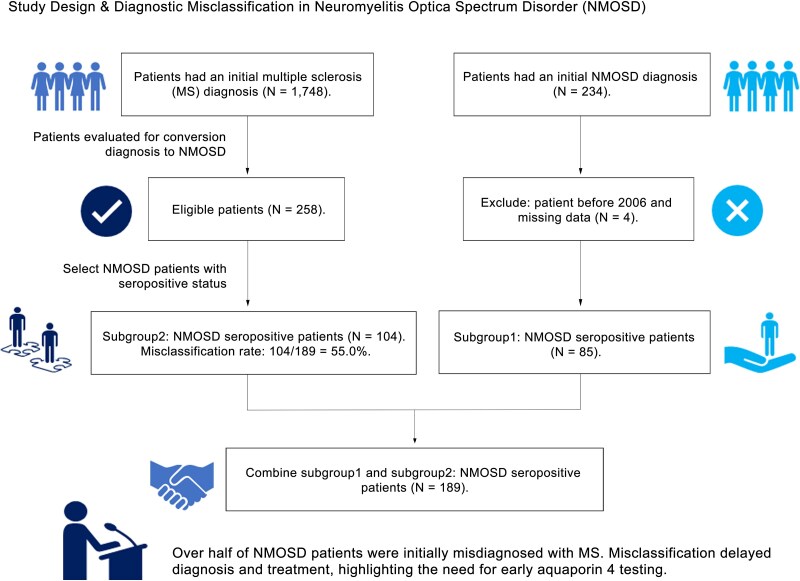

## Introduction

Neuromyelitis optica spectrum disorder (NMOSD) is a rare antibody-mediated inflammatory disease of the central nervous system. Previous studies on the incidence and prevalence of NMOSD have demonstrated ethnic differences in NMOSD, with a higher rate in non-Caucasian populations than in Caucasian populations.^1,2^ Overall, the worldwide incidence and prevalence rates of NMOSD range from 0.03 to 0.88 and 0.07 to 10.00 per 100 000 population, respectively.^3,4^

The typical mean age of onset of NMOSD varies from 29.2 to 45.7 years with a female predominance.^2^ Approximately 3–5% of patients have the paediatric form of NMOSD.^5^ Although the discovery of pathogenic autoantibodies to aquaporin 4 (AQP4) and the introduction of the International Panel for NMO Diagnosis (IPND) criteria have greatly improved diagnostic accuracy, the correct diagnosis of NMOSD is still frequently overlooked in clinical practice.^6,7^ One database study showed that 7.4% of NMOSD cases were initially diagnosed as multiple sclerosis (MS), demonstrating that the clinical and radiological criteria were difficult or inadequate to separate MS from NMOSD.^8^ Several factors may contribute to misclassification of NMOSD as MS, including the clinical presentations, the time elapsed before consulting a neuroimmunology specialist, and the method used to detect the AQP4 autoantibody.^9,10^ Given that NMOSD is a rare disease, misclassification of patients can lead to inaccurate estimates of its incidence and prevalence.

As pharmacological advances significantly reduce NMOSD relapses, updating precise incidence and prevalence data is crucial, especially in Taiwan, where well-designed epidemiological studies remain limited.^11,12^ This study aimed to determine the incidence, prevalence, and misclassification rate of NMOSD using a sophisticated study design. This was achieved through a retrospective cohort-based study on hospital-based surveillance data and national disease registry data over a long observational period. The purpose was to provide the epidemiological data from the national disease registry database.

## Materials and methods

### Data source

This study was a cohort analysis utilizing data from the Chang Gung Research Database (CGRD) and the Taiwan National Health Insurance Research Database (NHIRD). The CGRD is an extensive repository derived from the medical records of Chang Gung Memorial Hospital, the largest healthcare system in Taiwan. It provides a comprehensive set of clinical data, including diagnostic records, prescribed medications, and laboratory findings, such as anti-aquaporin-4 (AQP4) autoantibody test results.^13^ Using the same searching strategy in the CGRD cohort, we attempted to verify that our identified population was patients with NMOSD, and this method could be projected to the large national cohort (NHIRD). The NHIRD cohort consists of medical claim data from nearly the entire population of Taiwan, as described previously.^14,15^ Briefly, the NHIRD cohort includes comprehensive demographic information, information from clinical visits, diagnostic codes and details of prescriptions without the test results of anti-AQP4 autoantibodies.

### Study cohort

**Figure 1:**
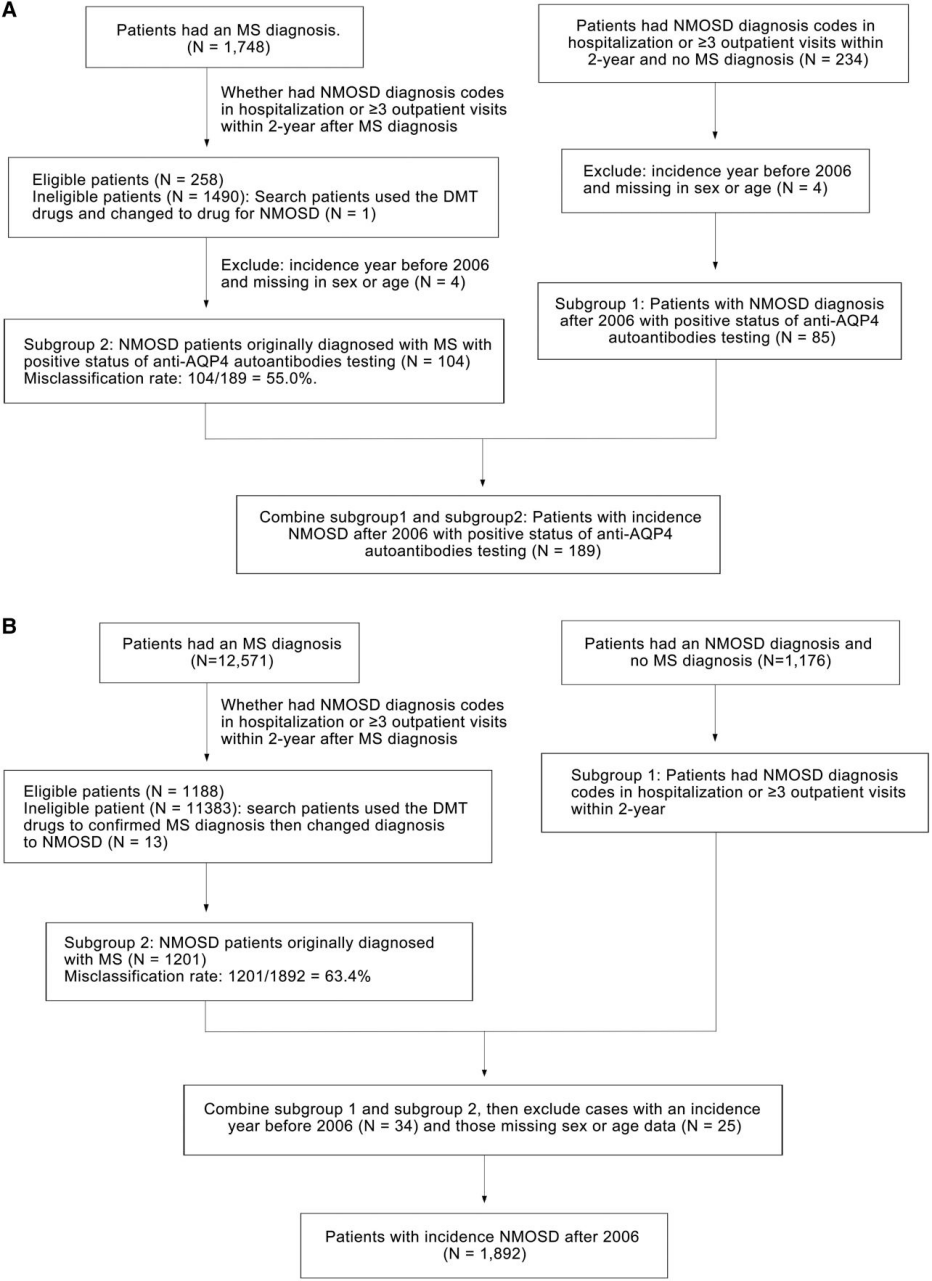
**Study cohort overview.** Flow chart of the study cohort in the (**A**) Chang Gung Research Database (CGRD) and (**B**) National Health Insurance Research Database (NHIRD).

The study cohort was derived from the Chang Gung Research Database (CGRD) for 2005–2021 and the Taiwan National Health Insurance Research Database (NHIRD) for 2005–2020. This cohort identification approach was adapted and modified from our previously published work on relapse patterns and comorbidities in NMOSD.^16^ NMOSD cases were defined using diagnostic criteria consistent with the revised Wingerchuk criteria (pre-2015) and IPND standards (post-2015).^6,17^ In addition, the National Welfare Bureau had a stringent policy to approve the diagnosis of rare diseases. Patients were required to meet The International Statistical Classification of Diseases and Related Health Problems (ICD) coding criteria combined with either hospitalization or multiple outpatient visits. Given the heterogeneity and diagnostic challenges of seronegative NMOSD, we focused exclusively on seropositive cases to ensure the reliability of epidemiological estimates in the CGRD cohort. In the NHIRD cohort, we can only use ICD diagnosis coding from the claimed dataset. Unlike the CGRD cohort, NHIRD does not contain AQP4 serostatus data. As a result, we could not distinguish seropositive from seronegative NMOSD cases in NHIRD, and the findings from the two cohorts should be interpreted accordingly. For misclassification analysis, individuals with a prior diagnosis of MS who were later diagnosed with NMOSD and treated with disease-modifying therapies (DMTs) were reclassified based on subsequent positive anti-AQP4 antibody results and receipt of NMOSD-specific treatment. The proportion of misclassification in NMOSD was calculated as the number of patients with a final diagnosis of NMOSD who were initially misdiagnosed with MS divided by the total number of patients with a final diagnosis of NMOSD. The patient selection process for both cohorts is summarized in Fig. 1.

### Clinical information and relapse rate

To characterize the clinical profile of NMOSD patients, demographic and treatment-related variables were extracted from both inpatient and outpatient medical claims. Patients were categorized into paediatric (<18 years) and adult (≥18 years) groups based on age at diagnosis. Anti-AQP4 antibody status was determined through enzyme-linked immunosorbent assays (ELISA) testing prior to 2018 and cell-based assays (CBA) thereafter, with seropositivity defined by at least one confirmed positive result during the observation window in the CGRD dataset.

Relapse episodes were operationally defined by the administration of acute-phase treatments—such as high-dose corticosteroids (minimum 3000 mg methylprednisolone over a period of 3–7 days), plasma exchange, or intravenous immunoglobulins—during hospital admission. Repeated treatments within a 30-day window were considered part of the same relapse event. The first such occurrence defined the index date for each patient.

Relapse frequency was quantified as an annualized relapse rate (ARR), calculated by dividing the total number of distinct relapses by each patient's cumulative follow-up time. Severity of disease activity was further stratified into ‘frequent’ (≥2 relapses within 2 years) and ‘high-frequency’ (≥2 relapses in 1 year) subgroups. Comorbid diagnoses were confirmed using ICD codes across multiple outpatient visits or inpatient admissions. Autoimmune and malignant comorbidities—such as systemic lupus erythematosus, Sjögren’s syndrome and cancer—were flagged regardless of their temporal relationship to the index date. The definition of relapses and categorization of comorbidities were adapted from our previous study on NMOSD comorbidity patterns.^16^

### Statistical analysis

The incidence and prevalence rates of NMOSD were calculated based on the index date of inclusion, defined as the first recorded diagnosis of NMOSD in the dataset after meeting the study criteria. In the CGRD cohort, the hospital incidence rate refers to the number of newly diagnosed NMOSD cases per 100 000 total hospital patients visiting annually. This reflects hospital-based trends and may be influenced by referral patterns. In contrast, the general incidence rate refers to cases per 100 000 in the overall population in NHIRD, providing a broader epidemiological perspective. The number of relapses and the ARR were estimated and reported using 3 years after NMOSD diagnosis as the cut-off point. We also categorized the ARR into four groups (< 1, 1 to <2, 2 to <3, and ≥3). All analyses were stratified by adult and paediatric form using age 18 years as the cut-off. For all analyses, differences in median values were tested using the Wilcoxon rank-sum test. The significance level was set at 0.05. All data analyses were performed with SAS 9.4 (SAS Institute).

### Institutional review board

This study was approved by the ethics review board of the National Health Research Institute of Chung Gang Memorial Hospital (approval numbers 201802146A0, 202201613B0, and 202201493A0C602A3).

## Results

### VIncidence and relapse rates of NMOSD in the CGRD cohort

A total of 485 patients with diagnostic codes for NMOSD were identified in the CGRD cohort. Of these, 466 patients (96.1%) had the adult form of NMOSD, while 19 patients (3.9%) had the paediatric form, based on claimed data. When we restricted the analysis to NMOSD patients with positive anti-AQP4 autoantibody results, 193 patients were identified. This subgroup included 189 adults (97.9%) and 4 paediatric patients (2.1%).

For the adult form of NMOSD, the mean confirmed age was 47.0 ± 15.1 years, with a female-to-male sex ratio of 20:1. Due to the small sample size, the paediatric form of NMOSD was excluded from further analyses. Among the adults, 32% had comorbidities, with Sjögren's syndrome being the most common, present in 21.2% of patients (Table 1).

**Table 1:** Demographic characteristics of NMOSD patients with a seropositive status of anti-AQP4 autoantibodies in the CGRD cohort from 2006 to 2021

Characteristics	Adult NMOSD patients
No. of patients	189
Confirmed age (years)^a^	
Mean ± SD	47.0 ± 15.1
Median (IQR)	47.6 (19.5)
Age at onset (years), mean ± SD	45.3 ± 14.4
Male, *n* (%)	9 (4.8)
Sex ratio (female/male)	20
Comorbidities, *n* (%)	61(32.3)
Malignancy	12 (6.4)
Systemic lupus erythematosus	12 (6.4)
Systemic sclerosis	1 (0.5)
Rheumatic arthritis	2 (1.1)
Sjogren's syndrome	40 (21.2)
Ankylosing spondylitis	0 (0.0)
Vasculitis	0 (0.0)
Autoimmune thyroiditis	4 (2.1)
Had hospitalization of NMOSD, *n* (%)	166 (87.8)
Had positive status of AQP4 autoantibody test, *n* (%)	189 (100)
Duration of follow-up^a^ (years), median (IQR), range	2.9 (1.1–6.0), 0.002–16.4
<5 years, *n* (%)	110 (58.2)
≥5 years, *n* (%)	79 (41.8)
No. of patients with relapses, *n* (%)	
Intensive relapses, *n* (%)	57 (30.2)
Extremely intensive relapses, *n* (%)	42 (22.2)

Frequent relapses: at least two relapses within a 2-year interval, and extremely frequent relapses were defined as at least two relapses within a 1-year interval.

MS, multiple sclerosis; AQP4, aquaporin 4. CGRD, Chang Gung Research Database; IQR, interquartile range.

^a^The age and disease duration do not follow normal distribution which are tested by Shapiro–Wilk tests.

The hospital incidence rate of NMOSD in patients with positive anti-AQP4 autoantibodies gradually increased from 0.08 per 100 000 persons in 2006 to 1.19 per 100 000 persons in 2021, peaking at 2.43 per 100 000 persons in 2017 (Supplementary Table 1).

The relapse events and ARR in the adult form of NMOSD from the CGRD cohort showed that the median number of relapses was 1 [interquartile range (IQR): 1–2], and the median ARR was 0.49 (IQR: 0.26–0.85). The ARRs within 3 years (median: 0.67, IQR: 0.33–1.00) were significantly higher than those beyond 3 years (median: 0.00, IQR: 0.00–0.27) in patients with positive anti-AQP4 autoantibody status by Wilcoxon rank-sum test (*P* < 0.01) (Table 2).

**Table 2:** Annualized relapse rates for adult patients with NMOSD in CGRD with seropositive status of anti-AQP4 autoantibodies

	NMOSD patients (*N* = 189)	Time after NMOSD diagnosis date		
		<3 Years	≥3 Years	*P*-Value^a^
No. of relapses				
Median (IQR)	1 (1, 2)	1 (1, 1)	0 (0, 1)	<0.001
Range	1–15	1–9	0–10	
Annualized relapse rates				
Median (IQR)	0.49 (0.26, 0.85)	0.67^b^ (0.33, 1.00)	0.00 (0.00, 0.12)	<0.001
Range	0.07–365.00	0.33–365.00	0.00–2.09	

^a^The differences between <3 years and ≥3 years for the number of relapses and annualized relapse rates were tested by the Wilcoxon rank-sum test.

^b^The difference of ARR within 3 years after NMOSD diagnosis was tested by the Wilcoxon rank-sum test between the adult and paediatric cohort, and the *P*-value was 0.278.

### The proportion of misclassification in the CGRD cohort

For the misclassification study, 1748 patients from the CGRD cohort were initially diagnosed with MS, of whom 104 patients were ultimately reclassified as having NMOSD with a positive status on anti-AQP4 autoantibody testing (Fig. 1A). These 104 patients represented 5.9% of those initially diagnosed with MS. The 104 out of 189 NMOSD patients (55.0%) were initially misdiagnosed as MS from the CGRD cohort. On the other hand, 85 patients had an initial diagnosis code of NMOSD and a positive anti-AQP4 autoantibody status without a diagnosis of MS. We compared the clinical characteristics of 104 NMOSD patients initially diagnosed with MS and subsequently reclassified to NMOSD and 85 NMOSD patients without a previous diagnosis of MS (Table 3). Patients with NMOSD misclassified as MS were found to have a younger age at onset, more frequent hospitalizations, a longer latency to perform AQP4 autoantibody testing, a longer follow-up period and a higher proportion of patients with frequent and extremely frequent relapses (all *P* < 0.05). Meanwhile, in the CGRD cohort, 33 patients initially diagnosed with MS had received at least 30 days of DMT and were eventually reclassified to the NMOSD diagnosis with positive anti-AQP4 autoantibody test results. NMOSD patients initially misclassified as MS who received DMT to treat MS compared with those initially diagnosed with NMOSD showed a significantly younger age at onset, more likely to be hospitalized, a higher malignancy rate, a lower Sjögren's syndrome rate, longer follow-up periods, and a higher proportion of patients with frequent and extremely frequent relapses (all *P* < 0.05), which were similar to the patients initially misclassified as MS group (Table 4).

**Table 3:** Demographic characteristics for NMOSD patients with and without an initial diagnosis of MS in patients with seropositive status of anti-AQP4 autoantibodies in CGRD cohort

Characteristic	Patients initially diagnosed with MS and then converted to NMOSD	Patients initially diagnosed as NMOSD	*P*-value^a^
No. of patients	104	85	
Confirmed age (years)			
Mean ± SD	45.0 ± 13.4	49.5 ± 11.9	0.05
Median (IQR)	46.1 (19.8)	50.4 (20.4)	0.03
Age at onset (years), mean ± SD	42.3 ± 13.1	49.0 ± 17.7	<0.01
Male, *n* (%)	2 (1.9)	7 (8.2)	0.08
Comorbidities, *n* (%)			
Malignancy	9 (8.7)	3 (3.5)	0.23
Systemic lupus erythematosus	5 (4.8)	7 (8.2)	0.38
Systemic sclerosis	0 (0.0)	1 (1.2)	0.45
Rheumatic arthritis	1 (1.0)	1 (1.2)	0.99
Sjogren's syndrome	20 (19.2)	20 (23.5)	0.48
Ankylosing spondylitis	0 (0.0)	0 (0.0)	N/A
Vasculitis	2 (1.9)	0 (0.0)	0.50
Autoimmune thyroiditis	3 (2.9)	1 (1.2)	0.63
Had positive status of AQP4 autoantibody test, *n* (%)	104(100)	85 (100)	1.00
Had hospitalization of NMOSD, *n* (%)	99 (95.2)	67 (78.8)	<0.01
Duration between index date to AQP4 autoantibody test (day), median (IQR)	20.0 (5.5–828.5)	12.5 (4.0–32.0)	<0.01
Duration of follow-up^a^ (years), median ± IQR (range)	5.6 ± 6.1 (0.2–16.2)	2.2 ± 3.7 (0.02–9.9)	<0.01
No. of patients with relapses			<0.01
Frequent relapses, *n* (%)	44 (42.3)	13 (15.3)	<0.01
Extremely frequent relapses, *n* (%)	32 (30.8)	10 (11.8)	<0.01

Frequent relapses: at least two relapses within a 2-year interval, and extremely frequent relapses were defined as at least two relapses within a 1-year interval.

MS, multiple sclerosis; AQP4, aquaporin 4. CGRD, Chang Gung Research Database; IQR, interquartile range.

^a^The difference of continuous variables was tested by the Wilcoxon rank-sum test for median and the student *t*-test for mean. The difference for categorical variables was tested by the Chi-square test.

**Table 4:** Demographic characteristics for NMOSD patients with an initial diagnosis of MS who received at least 30 days of DMT and those without an initial diagnosis of MS in CGRD

Characteristic	NMOSD patients initially diagnosed with MS and received at least 30 days of DMT	Patients initially diagnosed as NMOSD	*P*-value^a^
No. of patients	33	85	
Confirmed age (years)			
Mean ± SD	46.5 ± 11.9	49.5 ± 11.9	0.05
Median (IQR)	50.9 (20.3)	50.4 (20.4)	0.29
Age at onset (years), mean ± SD	39.9 ± 10.8	49.0 ± 17.7	<0.01
Male, *n* (%)	0 (0.0)	7 (8.2)	0.19
Comorbidities, *n* (%)			
Malignancy	6 (18.2)	3 (3.5)	0.01
Systemic lupus erythematosus	1 (3.0)	7 (8.2)	0.44
Systemic sclerosis	0 (0.0)	1 (1.2)	0.99
Rheumatic arthritis	0 (0.0)	1 (1.2)	0.99
Sjogren's syndrome	2 (6.1)	20 (23.5)	0.04
Ankylosing spondylitis	0 (0.0)	0 (0.0)	N/A
Vasculitis	1 (3.0)	0 (0.0)	0.28
Autoimmune thyroiditis	1 (3.0)	1 (1.2)	0.48
Had positive status of AQP4 autoantibody test, *n* (%)	33 (100)	85 (100)	1.00
Had hospitalization of NMOSD, *n* (%)	32 (97.0)	67 (78.8)	0.02
Duration of follow-up^a^ (years), median ± IQR (range)	8.8 ± 6.6 (0.4–16.2)	2.2 ± 3.7 (0.02–9.9)	<0.01
No. of patients with relapses			<0.01
Frequent relapses, *n* (%)	16 (48.5)	13 (15.3)	<0.01
Extremely frequent relapses, *n*(%)	16 (48.5)	10 (11.8)	<0.01

Frequent relapses: at least two relapses within a 2-year interval, and extremely frequent relapses were defined as at least two relapses within a 1-year interval.

MS, multiple sclerosis; AQP4, aquaporin 4. CGRD, Chang Gung Research Database; IQR, interquartile range.

^a^The difference of continuous variables was tested by the Wilcoxon rank-sum test for median and the student *t*-test for mean. The difference for categorical variables was tested by the Chi-square test.

### Incidence, prevalence, proportion of misclassification and relapse rates of NMOSD in the NHIRD cohort

**Figure 2:**
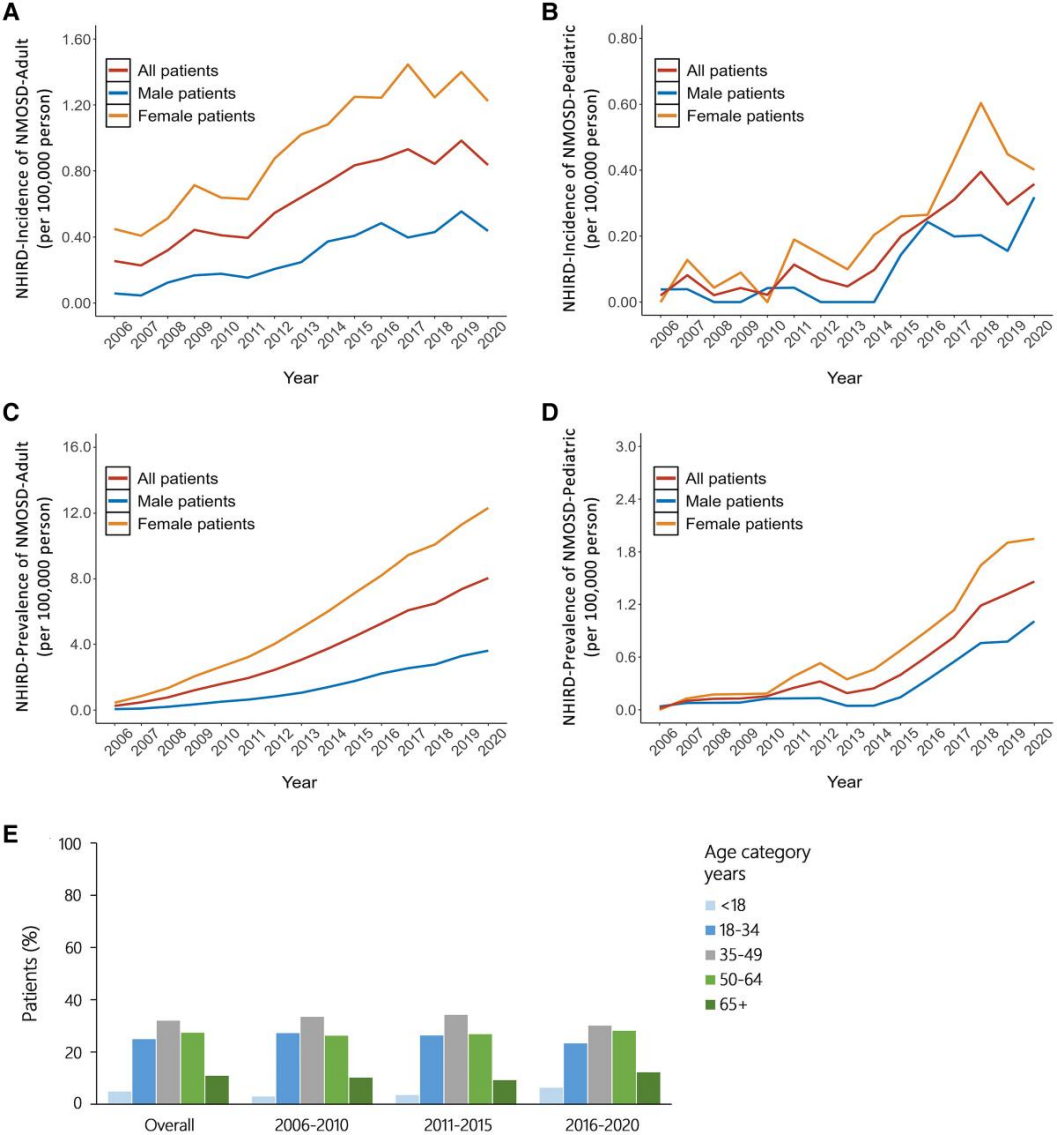
**NMOSD incidence and prevalence trends.** The incidence and prevalence of the adult (**A**, **C**) and paediatric (**B**, **D**) forms of NMOSD in the NHIRD and the percentage of NMOSD patients by incidence year and age group (**E**) from 2006 to 2020. The total number of NMOSD patients is 1800 for adults and 92 for paediatric patients in NHIRD.

A total of 1892 patients with a diagnosis of NMOSD fulfilled the inclusion criteria for the study cohort in the NHIRD cohort from 2006 to 2020, of whom 1800 patients had the adult form and 92 patients (4.9%) had the paediatric form of NMOSD. Because of the claimed data from NHIRD did not have the results of anti-AQP4 autoantibodies testing, we could not exclude the negative status of anti-AQP4 autoantibody testing patients in this dataset (Fig. 1B). In the NHIRD cohort, 1201 out of 1892 NMOSD patients (63.5%) were initially diagnosed as MS before being reclassified as NMOSD. These 1201 reclassified NMOSD patients represented 9.5% of the 12 571 patients who were initially diagnosed with MS. The mean confirmed age of those with the adult form of NMOSD was 45.4 ± 15.1 years, and the sex ratio (female/male) was 3.4; the mean confirmed age of those with the paediatric form was 12.5 ± 3.8 years, and the sex ratio (female/male) was 2.2. Supplementary Table 2 shows the crude incidence rate and prevalence rate of the adult and paediatric patients with NMOSD in the NHIRD cohort from 2006 to 2020. Regarding the adult form of NMOSD, there was a gradual increase in the crude incidence rate from 0.25 in 2006 to 0.84 in 2020 per 100 000 persons, which is higher than the CGRD cohort. A similar trend of increasing crude incidence rate was observed for the paediatric form, from 0.02 in 2006 to 0.32 in 2020 per 100 000 persons (Fig. 2A and B). Approximately 3–6% of patients had the paediatric form of NMOSD, and 9–12% of patients had an index day age greater than 65 years (Fig. 2E).

For the crude prevalence rate of NMOSD, there was a slowly increasing value from 0.25 in 2006 to 8.01 in 2020 per 100 000 persons for the adult form of NMOSD, while the paediatric form showed a similar tendency from 0.02 in 2006 to 1.46 in 2020 per 100 000 persons (Supplementary Table 2). Figure 2C and D depict the tendency of the crude incidence rate and prevalence rate of adult and paediatric forms of NMOSD from the NHIRD cohort.

**Figure 3:**
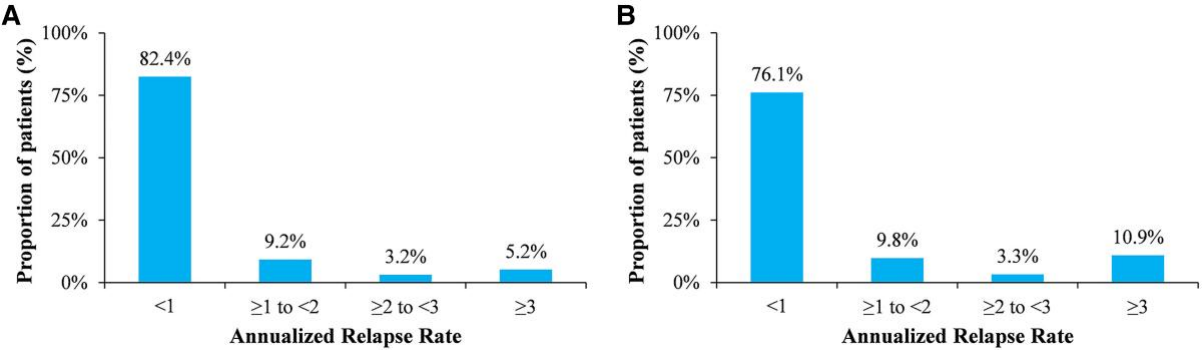
**Annualized relapse rate distribution.** Distribution of annualized relapse rates for adult (**A**) and paediatric (**B**) patients with NMOSD in the NHIRD. The experimental unit in this analysis is the individual patient. The NHIRD dataset includes *1800 adult patients* and *92 paediatric patients* diagnosed with NMOSD from 2006 to 2020. The ARR distribution reflects patient-level data, categorized by predefined relapse rate thresholds.

Relapse events and ARR in the NHIRD cohort showed that the median number of relapses was 1 (IQR: 1–2), and the median ARR was 0.36 (IQR: 0.16–0.75). In paediatric patients with NMOSD, the median number of relapses was 1 (IQR: 1–2), and the median ARR was 0.44 (range, 0.24–0.97). Significant differences in the number of relapses and ARRs were found within and beyond 3 years of diagnosis of NMOSD in both adults and children by the Wilcoxon rank-sum test (all *P* < 0.05) (Tables 5). According to the distribution of ARRs, among those with the adult form of NMOSD, approximately 82.4% had an ARR less than 1% and 5.2% had an ARR ≥ 3 (Fig. 3A), and among those with the paediatric form, approximately 76.1% had an ARR less than 1% and 10.9% had an ARR ≥ 3 (Fig. 3B).

**Table 5:** Annualized relapse rates for patients with NMOSD in NHIRD cohort

Adult form	NMOSD patients (*N* = 1800)	Time after NMOSD diagnosis date		
		<3 Years	≥3 Years	*P*-Value^a^
No. of relapses				
Median (IQR)	1 (1, 2)	1 (1, 2)	0 (0, 1)	<0.001
Range	1–39	1–17	0–29	
Annualized relapse rates				
Median (IQR)	0.36 (0.19, 0.75)	0.44 (0.33, 0.95)	0.00 (0.00, 0.09)	<0.001
Range	0.07–60.83	0.30–60.83	0.00–4.60	

Paediatric form	NMOSD patients (*N* = 92)	Time after NMOSD diagnosis date		
		<3 years	≥3 years	*P*-value^a^
No. of relapses				
Median (IQR)	1 (1, 2)	1 (1, 2)	0 (0, 0)	0.030
Range	1–20	1–12	0–13	
Annualized relapse rates				
Median (IQR)	0.44 (0.24, 0.97)	0.56 (0.33, 1.22)	0.00 (0.00, 0.00)	<0.001
Range	0.07–6.40	0.33–6.40	0.00–3.19	

NHIRD, National Health Insurance Research Database; IQR, interquartile range.

^a^The differences between <3 years and ≥3 years for the number of relapses and annualized relapse rates were tested by the Wilcoxon rank-sum test.

## Discussion

Several interesting and important conclusions can be drawn from the current study. First, our data showed a gradual increase in hospital incidence from 0.08 in 2006 to 1.19 in 2021 per 100 000 persons. A similar trend of incidence and prevalence from the NHIRD cohort was noted in both adult and paediatric forms of NMOSD. Second, we investigated the proportion of misclassification in NMOSD, which was 63.4% in the NHIRD cohort and 55.0% in the CGRD cohort. Third, we reported the comorbidities and median ARR in adults with NMOSD from CGRD and estimated the median ARR from the NHIRD cohort. The methodological strength of this study was regarded as more precise because our data were obtained from a tertiary medical centre database with more detailed clinical data and positive status of anti-AQP4 autoantibody tests than claimed data only, and a similar trend was found from the nationwide registry database. Moreover, this study employed a deliberate approach to identify NMOSD patients who were originally misdiagnosed with MS, enabling a more accurate and inclusive assessment of NMOSD surveillance in Taiwan compared to earlier investigations.^18^

### Prevalence and incidence rate in NMOSD

In the present study, the alteration in the hospital incidence rate of the CGRD cohort was examined, demonstrating an increase from 0.08 per 100 000 persons in 2006 to 1.19 per 100 000 persons in 2020. The NHIRD incidence rate is increasing over time from 0.25 per 100 000 persons to 0.84 per 100 000 persons. This phenomenon may be attributable to various factors, including increased awareness and updated diagnostic accuracy for NMOSD, such as the implementation of the IPND criteria in 2015 may led to more accurate classification of NMOSD cases, and increased diagnostic sensitivity and specificity from laboratory that is a shift from ELISA (pre-2018) to more sensitive CBA (post-2019) contributing to a higher detection rate.

In this study, the NHIRD cohort incidence rate is four times higher than the hospital incidence rate of the CGRD cohort in 2006, while relatively lower in 2020. This result may reflect that claims data from NHIRD, but limited AQP4 positive cases in the CGRD cohort in the early study period. The CBA test, which is widely available after 2019, and the CGRD cohort, which consists of patients from tertiary medical centres, may increase the number of patients in the hospital over time.

Several studies have shown ethnic differences in NMOSD prevalence, and Asians are at a higher risk.^19^ From one meta-analysis study, the worldwide prevalence rate was 1.54 per 100 000 persons, and the incidence rate was 0.15 per 100 000 persons.^3^ Our study indicated that the prevalence of NMOSD in adults and paediatric patients is similar to that in northern Japan (4.1 per 100 000 persons) and South Korea (3.56 per 100 000 persons).^20,21^ Interesting, a notable increase in paediatric cases since 2013 was related to the increasing awareness in the paediatric neurology field. Recently, a study reported that the age-standardized prevalence rate of the adult form of NMOSD gradually increased from 2003 to 2015, reaching 1.47 per 100 000 persons in 2015.^18^ Its relatively lower prevalence rate than that in the current study may have been caused by our population encompassing not only patients diagnosed with NMOSD but also those initially misclassified as MS.

Despite the increasing incidence of NMOSD, prevalence in the NHIRD cohort remained relatively stable. This phenomenon can be attributed to several factors. Firstly, NMOSD is associated with a high relapse-associated disability and mortality rate and a lack of adequate disease modifying therapy (DMT) in the early years, which limits long-term survival and thus prevents a proportional increase in prevalence.^22^ Second, NHIRD records only active patients based on medical claims; individuals who stop seeking medical care due to disease progression, mortality, or transition to palliative care may drop out of the dataset, leading to an underestimation of prevalence. Third, while improved diagnostic awareness and anti-AQP4 antibody testing have contributed to a rise in new diagnoses (higher incidence), these changes do not retroactively increase prevalence, as previously misclassified cases are not retrospectively counted as NMOSD in administrative datasets. These factors collectively contribute to the observed stability in NMOSD prevalence despite rising incidence.

### ARR for adult and paediatric forms of NMOSD

The median ARRs in the NHIRD cohort were 0.36 and 0.44 for the adult and paediatric forms of NMOSD, respectively. These results are similar to those of the CGRD cohort, showing that our hospital-based dataset can reflect and represent the nationwide dataset. A prior study reported a median ARR of 0.30 for the limited form of NMOSD, characterized by recurrent attacks at the same site, and 0.47 for the non-limited form.^12^ Additionally, a large-scale registry analysis from China indicated a median ARR of 0.4.^23^ These findings closely align with our results. Notably, our study extends previous research by providing the first analysis of paediatric median ARR in NMOSD within the Taiwanese population.^18,24^ Additionally, the ARR distribution revealed that over 70% of patients experienced an ARR below 1, while more than 10% had an ARR exceeding 2. In both adult and paediatric populations from the NHIRD and CGRD cohorts, the median ARR was notably higher during the first 3 years following diagnosis compared to later periods—an observation not previously documented in NMOSD studies from Taiwan. Recent studies have shown that the benign course of NMOSD is rare and that most disability in NMOSD is related to relapse rather than disease progression.^25,26^ Our findings highlight a greater likelihood of relapse within the initial 3 years following NMOSD diagnosis, underscoring the importance of initiating appropriate targeted treatments during this early phase to mitigate long-term disability. Prior research supports this approach, demonstrating that early use of disease-modifying therapies can effectively lower relapse frequency, and prompt management of acute episodes may help limit the progression of disability.^27,28^

### Proportion of misclassification in NMOSD

From previous literature, a patient-based dataset showed that 7.4–12% of patients with NMOSD were misclassified.^10^ The most frequent alternative diagnosis of NMOSD is MS.^8^ The seemingly high misclassification rate (55.0% in CGRD and 63.5% in NHIRD) reflects the proportion of NMOSD patients who were initially misdiagnosed as MS—a definition that differs from prior studies, such as Carnero Contentti *et al.*,^10^ where the misclassification rate is calculated using MS patients as the denominator. When applying the same approach, our MS-based misclassification rates are 5.9% (CGRD) and 9.5% (NHIRD), which are comparable to prior reports. Therefore, the difference stems from the definition rather than a true increase in diagnostic error. The value of our study is in demonstrating that, from the NMOSD cohort’s perspective, a large portion of patients were initially misdiagnosed, highlighting diagnostic challenges and opportunities for earlier intervention. Several factors may contribute to the misclassification of NMOSD, such as misinterpretation of clinical and neuroradiological findings and the availability of laboratory tests.^9^ Besides, the misclassification rate may be biased by the initial MS diagnosis based on one hospitalization criteria, which may overestimate the misclassification rate. Requiring at least three outpatient visits for MS would reduce potential overclassification, but at the cost of missing true NMOSD cases that were initially misdiagnosed due to evolving diagnostic criteria. Our findings elucidated crucial presentations among misclassified NMOSD patients. Notably, we observed a higher incidence of NMOSD among young individuals, particularly females, who were more likely to be hospitalized frequently. The presence of a higher relapse rate and longer duration of follow-up periods underscored the eventual correction of initial misclassification, emphasizing the pivotal role of clinical follow-ups and vigilant monitoring of disease evolution. One of the possible reasons for this misclassification is a significantly longer interval between first hospital visit and anti-AQP4 autoantibody testing (e.g. 20.0 days versus 12.5 days, *P* < 0.01). In addition, more frequent and extremely frequent relapses were found in this misclassified group. This highlights the urgent need for early anti-AQP4 autoantibody testing and a prompt, accurate diagnosis of NMOSD, as relapses are the major determining factor of disability in NMOSD.^29^ Furthermore, the misclassified group comprised a significant proportion of NMOSD, ranging from 55.0% in the CGRD cohort to 63.5% in the NHIRD cohort of the total NMOSD population. The misclassified group of patients could be excluded from our study if we only searched for the initial diagnosis codes of NMOSD patients, highlighting the importance of the search strategy to include NMOSD patients who were initially misclassified as having MS. Our study also showed that NMOSD patients who received at least 30 days of DMT had a significantly higher rate of hospitalization and relapse than NMOSD patients initially diagnosed. A previous study also showed that relapse induced by sphingosine-1-phosphate antagonists occurred within weeks of initiation in patients with NMOSD.^30^ Increased awareness of NMOSD through physician education may help to correctly differentiate NMOSD cases from the diagnosis of MS.

### Limitations

This study has several limitations. First, it was not a population-based study, and our results cannot be extrapolated to the whole population. Nevertheless, we utilized the dataset from our tertiary medical centre as a complementary tool to verify our findings and used stringent criteria to confirm the diagnosis of NMOSD, which is the best method to study rare disease cohorts. A key limitation is the absence of AQP4 serostatus data in NHIRD, making the differentiation between seropositive and seronegative NMOSD cases impossible. In contrast, CGRD includes only seropositive patients, ensuring more precise case identification. This discrepancy may impact comparability, affecting incidence and misclassification estimates. The hospital incidence is derived from CGRD, which captures patients diagnosed and treated at our tertiary medical centres; it may inherently reflect cases that were initially misclassified as MS and later were corrected according to NMOSD diagnostic criteria evolutions. This distinction is relevant, as it is consistent with our study’s objective of examining diagnostic misclassification at initial presentations. Nevertheless, both datasets indicate a rising NMOSD incidence in Taiwan. Second, we selected only seropositive NMOSD patients to improve our diagnostic accuracy. The contribution of clinical features and relapse rates of seronegative NMOSD was not considered in our study. Besides the delayed introduction of AQP4-IgG antibody testing, the incidence rates derived from CGRD may be underestimated in the earlier years. However, the increasing trend observed in our datasets aligns with increased clinical awareness and improved diagnostic accuracy. Moreover, we did not include anti-myelin oligodendrocyte glycoprotein antibody (MOG-Ab) because of low availability in our cohorts. In addition, the status of anti-AQP4 autoantibody testing in NHIRD was not available, which did not improve diagnostic accuracy in this cohort. Epidemiological results from NHIRD may be overestimated by our study. Thirdly, changes in the diagnostic criteria over the course of the study period may have influenced the misclassification rate, incidence and prevalence. The NMOSD diagnostic criteria evolutions before and after 2015 should be taken into consideration when interpreting misclassification rates. To account for evolving diagnostic criteria, future studies could stratify misclassification rates based on pre- and post-2015 diagnostic guidelines. Fourth, our ARR analysis is that it does not account for DMT initiation or treatment modifications, which can significantly impact relapse rates. Since DMT initiation was not included as a stratification factor, ARR trends should be interpreted cautiously, as they do not account for treatment effects. Future studies should incorporate DMT status to better understand its impact on relapse rates. We recognized that we could not perform a chart review to confirm the NMOSD diagnosis from the NHIRD cohort and the whole CGRD cohort in the current study. All these factors could influence the relapse rate and ARR in our study. Despite these limitations, the current study demonstrates more comprehensive data on the ARR, the vulnerable time for a higher relapse rate and epidemiologic information in NMOSD patients, which gives us a more accurate and insightful view of patients with NMOSD in Taiwan than previous studies.^24^

## Conclusion

Our study reports the clinical characteristics of NMOSD from our tertiary hospital database and the nationwide registry database using a sophisticated and complementary method based on selection criteria and validation. The incidence, prevalence rate, proportion of misclassification and ARR of adult and paediatric forms of NMOSD in Taiwan show ethnically related differences from those in Western countries. In addition, the median ARR significantly differed between cases less than and more than 3 years after NMOSD diagnosis, suggesting the necessity to treat NMOSD patients with more appropriate targeted therapy in the early years to prevent severe disability in the future.

## Supplementary Material

fcaf293_Supplementary_Data

## Data Availability

All data and codes generated used in this study will be available upon request to the corresponding author. The code used for statistical analysis to an online repository, which can be accessed at https://github.com/janelin0418/NMOSD.git.
