# Supplementary material for: Epidemiology and diagnostic challenges in neuromyelitis optica spectrum disorder in Taiwan: a hospital-based surveillance accompanied by a nationwide study
Source: Brain Commun. 2025 Aug 14;7(4):fcaf293. doi: 10.1093/braincomms/fcaf293 (PMC12392899; doi:10.1093/braincomms/fcaf293)
Supplement: fcaf293_Supplementary_Data [file fcaf293_supplementary_data.pdf]

Supplementary table 1. Incidence of NMOSD patients with seropositive status for anti-AQP4 autoantibodies testing in the CGRD cohort from 2006 to 2021.

Year	Incident cases (n)	Incidence of NMOSD per 100,000 persons (95% CI) in CGMH
2006	1	0.08 (0.00 to 0.25)
2007	2	0.17 (0.00 to 0.39)
2008	1	0.08 (0.00 to 0.24)
2009	5	0.40 (0.05 to 0.75)
2010	4	0.33 (0.01 to 0.65)
2011	7	0.56 (0.15 to 0.98)
2012	5	0.39 (0.05 to 0.73)
2013	13	1.00 (0.46 to 1.54)
2014	17	1.28 (0.67 to 1.89)
2015	11	0.81 (0.33 to 1.29)
2016	15	1.10 (0.54 to 1.65)
2017	33	2.43 (1.60 to 3.26)
2018	17	1.25 (0.66 to 1.85)
2019	21	1.50 (0.86 to 2.14)
2020	16	1.23 (0.63 to 1.83)
2021	17	1.19 (0.63 to 1.76)

Supplementary table 2. Incidence and Prevalence Rate (95% CI) of NMOSD in NHIRD cohort from 2006 to 2020.

(a) Adult

Calendar year and crude rate	Total	Gender		
		Male	Female	Sex ratio (Female/Male)
Crude incidence rate per 100,000 persons (95% CI)				
2006	0.25 (0.18 to 0.33)	0.06 (0.20 to 0.31)	0.45 (0.11 to 0.40)	7.71
2007	0.23 (0.16 to 0.30)	0.05 (0.18 to 0.27)	0.41 (0.09 to 0.36)	8.91
2008	0.32 (0.24 to 0.40)	0.12 (0.25 to 0.39)	0.51 (0.17 to 0.47)	4.14
2009	0.44 (0.35 to 0.54)	0.17 (0.36 to 0.53)	0.71 (0.27 to 0.62)	4.25
2010	0.41 (0.32 to 0.50)	0.18 (0.32 to 0.50)	0.64 (0.25 to 0.57)	3.60
2011	0.40 (0.30 to 0.49)	0.15 (0.31 to 0.48)	0.63 (0.23 to 0.56)	4.10
2012	0.55 (0.44 to 0.65)	0.21 (0.45 to 0.64)	0.88 (0.36 to 0.73)	4.24
2013	0.64 (0.53 to 0.76)	0.25 (0.54 to 0.74)	1.02 (0.44 to 0.84)	4.13
2014	0.73 (0.61 to 0.86)	0.37 (0.61 to 0.86)	1.08 (0.53 to 0.94)	2.90
2015	0.84 (0.71 to 0.96)	0.41 (0.71 to 0.96)	1.25 (0.61 to 1.06)	3.07
2016	0.87 (0.74 to 1.00)	0.48 (0.73 to 1.01)	1.25 (0.65 to 1.09)	2.57
2017	0.93 (0.80 to 1.07)	0.40 (0.81 to 1.06)	1.45 (0.70 to 1.17)	3.64
2018	0.84 (0.72 to 0.97)	0.43 (0.72 to 0.97)	1.25 (0.63 to 1.06)	2.90
2019	0.98 (0.85 to 1.12)	0.56 (0.84 to 1.13)	1.40 (0.76 to 1.21)	2.52
2020	0.84 (0.71 to 0.96)	0.44 (0.71 to 0.97)	1.23 (0.63 to 1.05)	2.80
Crude prevalence rate per 100,000 persons (95% CI)				
2006	0.25 (0.18 to 0.33)	0.06 (0.20 to 0.31)	0.45 (0.11 to 0.40)	7.71
2007	0.47 (0.37 to 0.57)	0.09 (0.41 to 0.54)	0.85 (0.28 to 0.67)	9.28
2008	0.77 (0.65 to 0.90)	0.20 (0.68 to 0.87)	1.34 (0.53 to 1.01)	6.60
2009	1.21 (1.05 to 1.37)	0.35 (1.09 to 1.33)	2.06 (0.92 to 1.50)	5.92
2010	1.59 (1.41 to 1.77)	0.51 (1.44 to 1.74)	2.64 (1.26 to 1.92)	5.18
2011	1.95 (1.74 to 2.15)	0.64 (1.78 to 2.11)	3.22 (1.58 to 2.31)	5.05
2012	2.45 (2.22 to 2.67)	0.83 (2.26 to 2.63)	4.02 (2.04 to 2.85)	4.87
2013	3.06 (2.81 to 3.31)	1.06 (2.85 to 3.27)	5.00 (2.61 to 3.51)	4.73
2014	3.74 (3.46 to 4.01)	1.40 (3.50 to 3.98)	6.00 (3.25 to 4.22)	4.29
2015	4.48 (4.18 to 4.77)	1.77 (4.21 to 4.74)	7.11 (3.95 to 5.00)	4.02
2016	5.26 (4.93 to 5.58)	2.22 (4.96 to 5.56)	8.17 (4.69 to 5.82)	3.68
2017	6.05 (5.71 to 6.40)	2.54 (5.73 to 6.37)	9.42 (5.45 to 6.66)	3.71
2018	6.47 (6.12 to 6.81)	2.77 (6.14 to 6.79)	10.07 (5.86 to 7.07)	3.64
2019	7.34 (6.97 to 7.70)	3.29 (6.99 to 7.69)	11.27 (6.70 to 7.98)	3.43
2020	8.01 (7.63 to 8.39)	3.61 (7.64 to 8.38)	12.28 (7.34 to 8.68)	3.41

(b) Pediatric

Calendar year and crude rate	Total	Gender		
		Male	Female	Sex ratio (Female/Male)
Crude incidence rate per 100,000 persons (95% CI)				
2006	0.02 (-0.02 to 0.06)	0.04 (-0.06 to 0.10)	0.00 (0.02 to 0.02)	N/A
2007	0.08 (0.00 to 0.16)	0.04 (0.00 to 0.16)	0.13 (-0.06 to 0.23)	3.27
2008	0.02 (-0.02 to 0.06)	0.00 (0.02 to 0.02)	0.04 (-0.06 to 0.11)	N/A
2009	0.04 (-0.02 to 0.10)	0.00 (0.04 to 0.04)	0.09 (-0.08 to 0.17)	N/A
2010	0.02 (-0.02 to 0.07)	0.04 (-0.06 to 0.11)	0.00 (0.02 to 0.02)	N/A
2011	0.11 (0.01 to 0.21)	0.04 (0.03 to 0.20)	0.19 (-0.07 to 0.30)	4.36
2012	0.07 (-0.01 to 0.15)	0.00 (0.07 to 0.07)	0.14 (-0.09 to 0.23)	N/A
2013	0.05 (-0.02 to 0.11)	0.00 (0.05 to 0.05)	0.10 (-0.09 to 0.19)	N/A
2014	0.10 (0.00 to 0.19)	0.00 (0.10 to 0.10)	0.20 (-0.10 to 0.30)	N/A
2015	0.20 (0.06 to 0.34)	0.14 (0.04 to 0.36)	0.26 (-0.03 to 0.43)	1.81
2016	0.25 (0.10 to 0.41)	0.24 (0.04 to 0.47)	0.26 (0.02 to 0.49)	1.09
2017	0.31 (0.13 to 0.49)	0.20 (0.12 to 0.51)	0.43 (0.01 to 0.61)	2.17
2018	0.40 (0.20 to 0.60)	0.20 (0.20 to 0.59)	0.60 (0.04 to 0.75)	2.98
2019	0.30 (0.12 to 0.47)	0.16 (0.12 to 0.47)	0.45 (-0.01 to 0.61)	2.89
2020	0.36 (0.16 to 0.55)	0.32 (0.10 to 0.61)	0.40 (0.06 to 0.66)	1.26
Crude prevalence rate per 100,000 persons (95% CI)				
2006	0.02 (-0.02 to 0.06)	0.04 (-0.06 to 0.10)	0.00 (0.02 to 0.02)	N/A
2007	0.10 (0.01 to 0.19)	0.08 (-0.01 to 0.21)	0.13 (-0.04 to 0.25)	1.64
2008	0.13 (0.03 to 0.23)	0.08 (0.01 to 0.24)	0.17 (-0.05 to 0.30)	2.18
2009	0.13 (0.03 to 0.23)	0.08 (0.01 to 0.24)	0.18 (-0.05 to 0.30)	2.18
2010	0.15 (0.04 to 0.27)	0.13 (0.01 to 0.30)	0.18 (-0.03 to 0.34)	1.45
2011	0.25 (0.10 to 0.40)	0.13 (0.10 to 0.40)	0.38 (-0.01 to 0.51)	2.91
2012	0.32 (0.15 to 0.49)	0.13 (0.17 to 0.47)	0.53 (0.01 to 0.64)	3.99
2013	0.19 (0.06 to 0.32)	0.05 (0.10 to 0.28)	0.35 (-0.07 to 0.45)	7.62
2014	0.24 (0.09 to 0.39)	0.05 (0.15 to 0.34)	0.46 (-0.06 to 0.54)	9.80
2015	0.40 (0.20 to 0.59)	0.14 (0.24 to 0.56)	0.68 (0.03 to 0.77)	4.71
2016	0.61 (0.37 to 0.85)	0.34 (0.36 to 0.86)	0.90 (0.18 to 1.04)	2.64
2017	0.83 (0.54 to 1.12)	0.55 (0.51 to 1.15)	1.13 (0.34 to 1.31)	2.07
2018	1.19 (0.84 to 1.53)	0.76 (0.80 to 1.57)	1.65 (0.60 to 1.77)	2.17
2019	1.32 (0.95 to 1.69)	0.78 (0.93 to 1.71)	1.91 (0.68 to 1.96)	2.45
2020	1.46 (1.07 to 1.85)	1.01 (1.01 to 1.91)	1.95 (0.80 to 2.11)	1.94

N/A, Not applicable
